# Social Media and HIV: A Systematic Review of Uses of Social Media in HIV Communication

**DOI:** 10.2196/jmir.4387

**Published:** 2015-11-02

**Authors:** Tamara Taggart, Mary Elisabeth Grewe, Donaldson F Conserve, Catherine Gliwa, Malika Roman Isler

**Affiliations:** ^1^ Department of Health Behavior University of North Carolina at Chapel Hill Chapel Hill, NC United States; ^2^ Institute for Global Health and Infectious Diseases University of North Carolina at Chapel Hill Chapel Hill, NC United States; ^3^ Department of Social Medicine University of North Carolina at Chapel Hill Chapel Hill, NC United States; ^4^ North Carolina Translational and Clinical Sciences Institute University of North Carolina at Chapel Hill Chapel Hill, NC United States

**Keywords:** HIV, social media, communication

## Abstract

**Background:**

Social media, including mobile technologies and social networking sites, are being used increasingly as part of human immunodeficiency virus (HIV) prevention and treatment efforts. As an important avenue for communication about HIV, social media use may continue to increase and become more widespread.

**Objective:**

The objective of this paper is to present a comprehensive systematic review of the current published literature on the design, users, benefits, and limitations of using social media to communicate about HIV prevention and treatment.

**Methods:**

This review paper used a systematic approach to survey all literature published before February 2014 using 7 electronic databases and a manual search. The inclusion criteria were (1) primary focus on communication/interaction about HIV/acquired immunodeficiency syndrome (AIDS), (2) discusses the use of social media to facilitate communication, (3) communication on the social media platform is between individuals or a group of individuals rather than the use of preset, automated responses from a platform, (4) published before February 19, 2014, and (5) all study designs.

**Results:**

The search identified 35 original research studies. Thirty studies had low or unclear risk of at least one of the bias items in the methodological quality assessment. Among the 8 social media platform types described, short message service text messaging was most commonly used. Platforms served multiple purposes including disseminating health information, conducting health promotion, sharing experiences, providing social support, and promoting medication adherence. Social media users were diverse in geographic location and race/ethnicity; studies commonly reported users aged 18-40 years and users with lower income. Although most studies did not specify whether use was anonymous, studies reported the importance of anonymity in social media use to communicate about HIV largely due to the stigma associated with HIV. The ability to share and receive information about HIV was the most commonly reported benefit of social media use and the most common challenges were related to technology. Measures of frequency of use, satisfaction, and effects of use varied across studies.

**Conclusions:**

Using social media to bridge communication among a diverse range of users, in various geographic and social contexts, may be leveraged through pre-existing platforms and with attention to the roles of anonymity and confidentiality in communication about HIV prevention and treatment. More robust research is needed to determine the effects of social media use on various health and social outcomes related to HIV.

## Introduction

Social media platforms, including mobile technologies and social networking sites, are being used increasingly as part of human immunodeficiency virus (HIV) prevention and treatment efforts [1-4]. Importantly, social media provides users with the opportunity to generate, share, and receive information through bi- and multidirectional exchanges, which may transcend geographic borders and provide an opportunity for anonymity [5-8]. Although stigma and cultural context may prevent people living with HIV/acquired immunodeficiency syndrome (AIDS) and at-risk populations from accessing in-person HIV prevention and treatment initiatives [9,10], social media can offer a neutral platform for engagement [11]. For example, individuals can seek and share information about specific prevention strategies [4,12], engage in dialog about HIV research [13], and leverage support for issues such as medication adherence and emotional coping for living with HIV [14-17]. Additionally, the increased social support provided by social media has been shown to improve treatment adherence and access to HIV testing and prevention services [18,19], and assist with coping with HIV-related stigma [17]. Social media use among key populations affected by the HIV epidemic, including men who have sex with men (MSM) [20-24], racial and ethnic minorities [25], and adolescents [26,27] is increasing, and studies demonstrate that these groups use social media to form social ties, access health information and emotional support, and build a sense of community with peers [28-32]. The social media activity of these groups can be leveraged to facilitate community engagement [33-35], which has been identified by the Joint United Nations Programme on HIV/AIDS (UNAIDS) as a critical component in HIV control efforts [36].

The widespread use of social media represents an important avenue for communication about HIV [37,38]. Further, as the globalization of HIV and its presence in more geographically distant and underserved communities increases, social media provides an opportunity to extend the reach of HIV prevention and treatment efforts. Currently lacking, however, is a thorough examination of the various users, platforms, and approaches to using social media to communicate about HIV. The objective of this paper is to address this gap by presenting a comprehensive systematic review of the current published literature on the design, users, benefits, and limitations of using social media to communicate about HIV prevention and treatment. This examination can inform critical next steps to ensure appropriate use of social media to reach and engage those most affected by HIV in their local milieu.

## Methods

### Search Strategy

This review paper followed the Preferred Reporting Items for Systematic Reviews and Meta-Analyses (PRISMA) guidelines [39] and used a systematic approach to retrieve relevant research studies. The review included all study designs and study methods, including qualitative, quantitative, and mixed-methods studies. The literature search was conducted on February 19, 2014, using the following 7 electronic databases: Cochrane Library, Cumulative Index to Nursing and Allied Health Literature, Dissertations, Embase, PsycINFO, PubMeb Central, and Web of Science. The searches were performed using the following defined search terms: (HIV OR “Human immunodeficiency virus” OR hiv infection* OR hiv infections[mesh] OR acquired immunodeficiency syndrome[mesh] OR “acquired immunodeficiency syndrome”) AND (“online community” OR “online communities” OR “virtual community” OR “virtual communities” OR Social Media[mesh] OR “social media” OR “Web 2.0″ OR “social medium” OR Social Networking[mesh] OR Social network*) AND (technolog* OR mobile* OR Internet OR online OR Blogging[mesh] OR blog* OR weblog* OR microblog* OR micro-blog* OR Twitter OR tweet* OR “mobile apps” OR “mobile app” OR “mobile applications” OR “mobile application” OR “online forum” OR “online forums” OR “bulletin board” OR “bulletin boards” OR “message board” OR “message boards” OR Skype OR instant messag* OR text messag* OR texting OR text messaging[mesh] OR YouTube OR Flickr OR Facebook OR LinkedIn OR MySpace OR SecondLife OR “Second Life” OR Listserv OR listserve OR “mailing list” OR “mailing lists” OR podcast* OR webcast* OR wiki*). Manual reference searches of identified systematic reviews were also completed.

### Selection Criteria

The retrieved articles were screened for relevance, duplication, and the selection criteria. The inclusion criteria were (1) primary focus on communication/interaction about HIV/AIDS; (2) discusses the use of social media to facilitate communication (social media was defined as platforms allowing for bi- or multidirectional exchange, including blogs, discussion boards, Facebook, etc); (3) communication on the social media platform was between individuals or a group of individuals rather than the use of preset, automated responses from a platform; (4) all literature published before February 19, 2014; and (5) all study designs. The exclusion criteria were (1) the focus on communication/interaction about HIV/AIDS was limited to study implications; (2) not in English; (3) commentary, letters to the editor or opinion pieces, protocols, and feature articles (ie, narrative-style journalistic pieces); (4) primary focus on marketing or advertising; (5) studies in which the social media platform was used for recruitment only; and (6) social media platform was used for data collection purposes only.

We completed title, abstract, and full-text review to identify all studies meeting inclusion and exclusion criteria. Three researchers (MRI, TT, CG) independently screened article titles for inclusion in abstract review. Next, working in pairs, the full research team independently reviewed and evaluated all retrieved abstracts and full texts, and reached consensus on the inclusion for the analysis. The interrater reliability between reviewers was 0.90, indicating strong agreement. Discrepancies were discussed within each dyad until consensus was reached; if no consensus was reached, the article underwent review by the full research team until consensus was reached. Studies excluded during the full-text review stage and their reasons for exclusion are listed in Multimedia Appendix 1.

### Data Extraction

Data were extracted using a set of 58 defined fields related to the design of the social media platform, social media user characteristics, use of the social media platform, benefits and disadvantages of using the social media platform, and study outcomes. Working in pairs, the research team independently extracted data from each article and then reconciled their responses to ensure consistency.

### Quality Assessment

Two members of the research team (TT, MG) conducted a quality assessment of the 35 included studies using a checklist tool for assessing quality in observational studies [40]. The 6 domains used to assess risk of bias included (1) methods for selecting study participants, (2) methods for measuring exposure and outcome variables, (3) design-specific source of bias, (4) method of control confounding, (5) statistical methods, and (6) other biases (including conflict of interest and disclosure of funding sources). For each study, the quality of each of these 6 items was categorized as low risk (+), high risk (-), or unclear (?) as recommended by the Cochrane Collaboration [41]. We added an additional category of not applicable (N/A).

## Results

**Figure 1:**
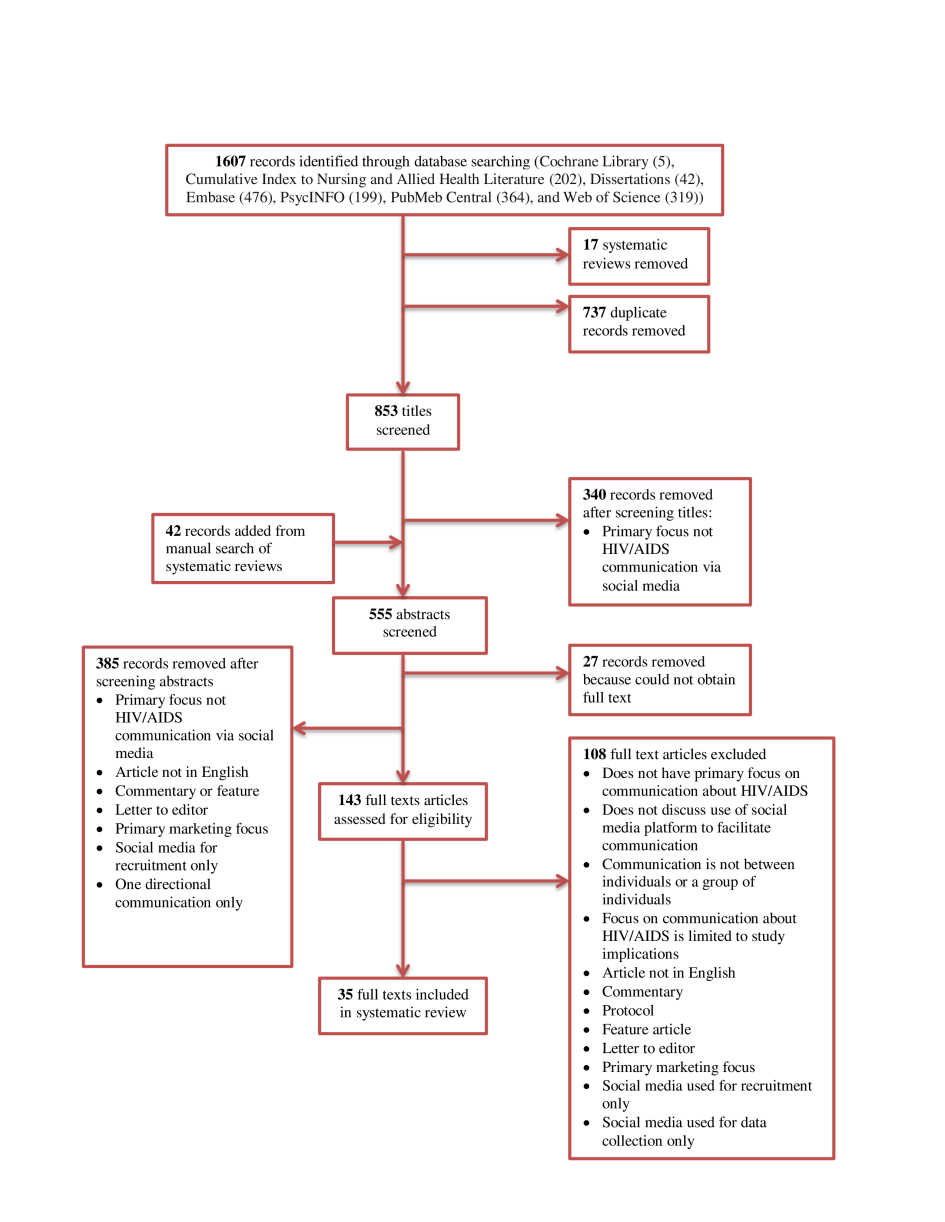
PRISMA diagram describing study selection process.

In all, 35 selected studies [12-18,42-69] met the inclusion criteria (see PRISMA diagram; Figure 1). Nine studies used qualitative research methods [13,14,16,44,45,50,53,54,61], 11 studies used quantitative research methods [12,15,42,46,48,49,51,52,56,63,66], and 15 studies used mixed methods [17,18,43,47,55,57-60,62,64,65,67-69]. The 35 included studies were summarized by study method, type of social media platform, participants/sample type and sample size, and topic(s) of discussion (see Multimedia Appendix 2). The most commonly described social media platforms to facilitate discussion around HIV were short message service (SMS) text messaging [15,45,47-49,52,55,66,67], discussion boards or forums [13,14,16,43,44,56,64,67,69], and social networking sites (eg, Facebook) [12,46,48,53,59,63,68] (the full list of social media platforms is provided in Table 1). The studies included a range of social media users, including the general public, people living with HIV/AIDS (PLWHA), and/or health professionals. Social media platforms discussed in the studies included a variety of communication features, such as discussion facilitators, directed or guided communication, and chat features. Studies reported various purposes for HIV communication on the social media platform, such as disseminating health information and/or promoting health [12,45,46,48,51,56-59,61-64,67,68], sharing thoughts and experiences [17,42,43,50,53,54,66], providing social support [12,14,16,57,64], and promoting medication adherence [15,47,49,55,65]. Topics of discussion included a range of issues related to HIV prevention (eg, skills and strategies to reduce risk), treatment (eg, medication adverse effects and adherence), coping (eg, disclosure, addressing stigma), and access to resources (eg, HIV services, online resources).

**Table 1:** Social media platforms used in selected studies (N=35).

Social media tool^a^	Studies
Blogs (n=4)	Adam et al (2011) [42], Eastham (2011) [50], Kvasny & Igwe (2008) [54], Strand (2011) [17]
Discussion forum/board (n=9)	Baelden et al (2012) [43], Brennan et al (1991) [44], Coursaris & Liu (2009) [14], Desouza & Jyoti Dutta (2008) [13], Lou et al (2006) [56], Mo & Coulson (2008) [16], Rothpletz-Puglia et al (2013) [64], Yamauchi 2010 [67], Zhuang & Bresnahan (2012) [69]
SMS text messaging (n=9)	Broaddus & Dickson-Gomez (2013) [45], Dean et al (2012) [47], Divecha et al (2012) [48], Dunbar et al (2003) [49], Hightow-Weidman et al (2014) [52], Horvath et al (2013) [15], Lester et al (2010) [55], Wicks et al (2010) [66], Yamauchi (2010) [67]
Social networking site (n=7)	Bull et al (2012) [46], Divecha et al (2012) [48], Hildebrand et al (2013) [53], Ko et al (2013) [12], Pedrana et al (2013) [59], Rice et al (2012) [63], Young & Jaganath (2013) [68]
Social networking site to private correspondence (n=3)	Feldacker et al (2011) [51], Hightow-Weidman et al (2014) [52], Young & Jaganath (2013) [68]
Video (n=3)	Desouza & Jyoti Dutta (2008) [13], Leon et al (2011) [18], Skrajner et al (2009) [65]
Chat (n=5)	Leon et al (2011) [18], Moskowitz et al (2009) [57], Pavlescak (2007) [58], Rhodes (2004) [61], Rhodes et al (2010) [62]
Conferencing (n=1)	Reid et al (2012) [60]

^a^ Some studies used multiple platforms.

### Quality Assessment

Among the 35 included studies, 5 were at low risk for all 6 methodological quality items (see Multimedia Appendix 3). The remaining 30 studies were at high or unclear risk of at least one of the bias items; none of the studies were at high risk for all bias items.

### Characteristics of Social Media Platform Users

The characteristics of social media users in the selected studies were diverse, representing a range of groups and populations. An assessment of the use of each type of social media platform by user characteristics did not produce a consistent trend. As such, the following results are across all types of social media platforms.

#### Geography

A total of 31 studies provided information on the location of social media users and 4 [14,17,45,58] did not provide this information. Seventeen studies described users located in the United States [15,44,46,48-52,54,57,61-65,68] and/or Canada [42,50]. Eight studies described users located in a region other than the United States or Canada [12,18,43,47,55,56,67,69], most commonly South Africa [43,47,67] and China [56,69]. Four studies described a global focus with users located in both the United States or Canada, and other countries [16,59,60,66]. Two studies [13,53] described an unrestricted geographic focus.

#### Sexual Orientation and HIV Status

A total of 12 studies reported social media users’ sexual orientation, which included heterosexual, homosexual, bisexual, and unsure or questioning [15,18,44,45,52,57,58,61-63,65,68]. All 12 studies included homosexual participants and 2 also included unsure or questioning participants [57,61]. Seventeen studies reported the HIV status of social media users [12,15-18,44,47,49,50,52,55,57,61,62,64-66]. These studies reported a range of study populations, including samples that were HIV-positive only, HIV-positive and HIV-negative, and unknown HIV status. Ten studies were exclusively for HIV-positive users, meaning only users who identified as HIV-positive could access the social media platform [15,18,44,47,49,50,55,64-66]. None of the included studies were exclusively for HIV-negative users.

#### Age and Gender

In all, 24 studies reported social media users’ age, ranging from adolescents to older adults aged 61 years and older [12,15,18,44-50,52,53,55-59,61-63,65-68]; most of these studies included users aged 18 to 40 years [12,18,44-50,52,53,55,57-59,61-63,65,67,68]. Twenty-six studies reported social media users’ gender [12,15-18,42-50,52,55,56,59,61-68]; 7 studies only included males [12,15,16,42,61,62,68] and 2 only included females [47,64]. Of the 17 studies that included both male and female participants, 11 had a majority of male participants [17,18,44,49,50,52,56,59,63,66,67].

#### Race

In all, 18 studies reported social media users’ race or ethnicity including, but not limited to, black, non-Hispanic white, Latino, and Asian [15,44-46,48-50,52,54,57,61-68]. Among the 17 studies in the United States and Canada reporting race, 5 reported more black social media users than non-Hispanic whites or other racial or ethnic minorities [45,48,52,54,65]. One of the 15 international studies reported users’ nationality [67]; the remaining 14 studies did not report users’ race, ethnicity, or nationality.

#### Socioeconomic Status

Reporting of social media users’ socioeconomic status (SES) varied across studies. Twenty-five studies did not report users’ income or employment status; of the remaining 10 studies, 6 studies reported users’ income [48,55,56,62,65,68] and 4 studies reported employment status [15,18,47,64]. Studies reporting employment status included users who were unemployed, employed full- or part-time, retired, and/or disabled. Although social media users’ income ranged from less than US $10,000 to US $80,000 a year, 5 of 6 studies reporting users’ income included low-income users reporting less than US $10,000 a year [48,55,62,65,68]; 1 study was not in the United States, whereas the other 4 were in the United States. Thirteen studies reported users’ current education and highest level of education attained, which ranged from high school or less to postcollege [12,15,18,43,44,47,55,56,60,62,64,65,68].

### Design of Social Media Platforms

All studies reported on some aspect of the social media platform, such as whether the platform required disclosure of identity and how users were engaged on the platform.

#### Process of Engagement

In all, 6 studies created new social media platforms [15,18,42,43,53,56]; the remaining studies used pre-existing platforms (eg, Facebook, YouTube, or SMS text messaging systems). Twenty-two studies reported that users were already accessing the platform before engagement for study purposes [13,14,16,17,45-48,50-52,54,55,57,58,61-63,66-69] and 10 studies reported that users started using the platform for the purpose of the study [15,18,42-44,49,53,56,64,65]. Social media facilitators included researchers [15,18,43,44,47,49,53,55,56,59,65,68]; nonresearchers, such as community members and program staff [13,14,16,17,42,45,46,48,50,54,58,61,63,66,67,69]; and both researchers and nonresearchers [12,51,52,57,60,64].

#### Communication on Social Media Platform

The most common form of communication on social media platforms was described as communication between users [13-16,18,43-48,50,53,54,56,59,64,66-69] followed by communication between users and staff/professionals (eg, clinicians, counselors, health educators, or health professionals) [15,18,44,47,49,51,52,55-62,65,67]. Communication between individuals (ie, one-to-one messaging) was more common between staff and users, and communication between groups of individuals (ie, posting for a group to read and respond to) was more common among users alone.

#### In-Person Components

Offline or in-person components that served a complementary or related programmatic purpose to the social media platform were reported in 11 studies [18,42,44,47,51-53,55,63,64,67]. Of these, 8 studies described complementary services being provided in-person, with the most common services being clinical care, counseling, or testing [18,44,47,51,55]. Additionally, a study described how social media users attended in-person workshops to help inform the process of online engagement [63] and 2 studies had options for individuals to be part of a content-equivalent offline program [53,64].

#### Disclosure of Identity During Platform Use

Most studies [12-17,42,45,46,48,51,53,54,56,58-64,66-69] did not clarify whether communication was anonymous or if social media users communicated using their real name or any other identifiable information. Two studies [44,50] indicated that users had the option to communicate using their real names. Five studies [18,49,52,55,65] stated that users communicated using their real names or faces on the platform. Two of these studies described videophone [65] or webcams [18] as components of the social media platform, and 3 of the studies [49,52,55] involved clinicians or staff contacting individuals for whom they knew the identity.

### Benefits of Social Media Use

The most common benefits to using social media to communicate about HIV that studies reported were (1) access to information, (2) enhanced ability to communicate, (3) having an anonymous identity, (4) a sense of social and emotional support, (5) establishing a virtual community, and (6) geographical reach. For most studies, benefits were perceived by the target groups/populations. For other studies, the researchers reported on their perceptions of benefits or challenges related to the social media use.

The ability to receive and share information was reported as a benefit in 12 studies. Studies described users appreciating the ease and convenience of accessing information related to HIV care, treatment, and prevention through social media. For example, users in 4 studies [14,15,43,66] reported being able to receive information online from other PLWHA about disease management. Another study [13] explored how social media users interested in HIV research in India were able to share information with other local and global users engaged in the same issues. One study [48] described how teens use their phones to find medical information and share information about HIV and other sexually transmitted infections on social media. Social media users in the remaining 6 studies [12,50,54,56,57,64] reported varied benefits, including access to an alternative, nontraditional source of information about HIV prevention and testing.

There were 9 studies reporting enhanced communication as a benefit. Users stated that social media provided them with an alternative, not in-person, way to communicate about sexual health, HIV testing, and condom use with peers, health professionals, and sexual partners. For example, in one study [44], PLWHA reported a benefit from being able to use social media to communicate with health professionals without leaving their homes. Social media users in 7 studies [12,45,47,59,63,67,68] including adolescents, noted that social media platforms such as SMS text messaging and Facebook allowed them to communicate about topics that they felt uncomfortable discussing in-person, such as condom use and HIV testing. In another study [54], a group of black American bloggers reported that the use of social media, specifically blogging, opened channels for communication about HIV, a topic they believed was underdiscussed within the black community.

Another benefit users reported was the anonymous nature of the social media platforms. Users in 6 studies [14,43,44,47,50,62] mentioned that the anonymity on social media platforms helped to decrease stigma, fear, and discrimination around HIV and allowed participants to tell personal stories about their sexual orientation and HIV status in a manner they would not with friends, family members, or sexual partners offline. The other 2 studies reported that the anonymity of the website allowed adolescent users to seek HIV prevention information [67] and MSM participants to successfully engage in the intervention [61].

Another benefit users reported from engaging in an online community was a sense of social and emotional support [14,16,17,44,47,50,54,67]. More specifically, adolescents, PLWHA, and MSMs reported experiencing a sense of community from engaging with others through social media [12,15,54,63]. Lastly, the ability for health care workers to reach patients and community members to engage with one another, regardless of geographical location, was reported as a benefit of using a social media platform to communicate about HIV [13,18,47,48,50,68].

### Disadvantages of Social Media Use

The most common disadvantages to using social media to communicate about HIV prevention and treatment that studies reported were related to (1) technology barriers, (2) cost, (3) lack of physical interaction, and (4) lack of privacy.

Technological barriers were reported in 12 studies and included users’ problems with poor Internet connection [44,49,56,60,61], insufficient access to computers [43,54], lack of technical help [62,67], poor quality of video and audio transmissions [65], low information technology literacy [60], and technical “glitches” [15]. Ten studies reported the cost of social media equipment (ie, personal computers) [18,44,47,65], Internet access [47,55,67], and human resources [52,55,62] as barriers.

Lack of physical interaction was a disadvantage reported in 6 studies [44,50,56,61,66,67] and included limitations in the amount of support health professionals can offer online. Additionally, the absence of verbal or nonverbal cues and the lack of transparency prevented some health professional users from being able to tailor their services and support to specific users. Other recurring disadvantages included lack of privacy and confidentiality [15,47,48,59,61], which prevented participants from sharing personal information out of fear that their information would not be protected. Less frequently cited disadvantages included lack of interest [59-61] and lack of time to communicate with other social media users [43].

### Outcomes of Social Media Use

The most common outcomes of social media use reported were the (1) frequency of social media use, (2) user satisfaction, (3) type of information shared, and (4) effects of social media use.

A total of 19 studies measured frequency of use in a variety of ways. The range of methods used to measure frequency of use included the number of days the social media platform was used [44]; number of visitors [42,46]; number of videophone calls [65] or video viewings [54,59,63]; number of instant message counseling sessions [57]; and number of conversations [58,62,68], messages [49,52,67], posts [12,15,16], attendance [60], and/or hits on the study website [56].

Eight studies measured users’ satisfaction with the platform by assessing acceptability [15,18,49], usefulness [66], quality [60], and access [44] on a scale of high, medium, and low satisfaction. Across these studies, users rated their satisfaction with the various social media platforms as high and reported that they found the platforms simple to use [47,49]. Users also reported the platforms provided access to a diverse group of users and that they would recommend the platform to their friends [55].

Five studies measured the type of information shared on the platforms (eg, access to HIV testing and safer sex strategies) and 4 studies reported that most of the messages contained informational support [13,14,54,63] followed by emotional and social network support [16]. Six studies measured the effects of social media use and found an increase in HIV knowledge [47], HIV-related discussions with online friends [12], number of people seeking HIV testing services [51], and new HIV-positive patients identified [52] along with improved medication adherence among HIV-positive participants [15,49].

## Discussion

Our review yielded 35 studies that used social media to communicate about HIV. Social media has been shown to facilitate discussion and information exchange on a range of health issues [70]. To our knowledge, this is the first review of the current landscape and users of social media to communicate about HIV exclusively. Our findings illustrate the following: (1) the value of using pre-existing social media platforms, (2) the diversity among the characteristics of users, and (3) the importance of the role of anonymity on the platform. Consideration of these findings will help extend the field of social media, specifically when related to communication about HIV prevention and treatment.

Most studies use pre-existing social media platforms (eg, a Facebook page) rather than creating new platforms. There may be a number of benefits to using pre-existing social media platforms to communicate about HIV, which might explain their widespread use. First, developing new social media platforms may be costly or resource intensive; some of these costs may be mitigated by using pre-existing platforms. Second, communicating through pre-existing platforms may decrease barriers associated with end-users learning new social media technology. Lastly, users’ familiarity with the platform and connection to other existing users may facilitate more open communication as social media ties increase and users form new virtual communities [71,72]. However, despite the potential ease of using pre-existing platforms, health care professionals in some of the selected studies reported limitations in their ability to form relationships with social media users in comparison to face-to-face interaction. This limitation may have an effect on user satisfaction and use of the platform, which is a matter of concern given the recent increase in digital interactions within health care [73]. Therefore, providing training and support to both users and facilitators (eg, health care professionals) is critical when implementing social media initiatives to mitigate technological or other usage barriers.

Characteristics of social media platform users communicating about HIV within our selected studies span a wide range of geographic locations, sexual orientations, ages, genders, races, and SES. This is encouraging, given the diverse and global nature of the HIV epidemic, and supports other studies that show the high acceptability and use of social media across diverse groups [25,38,74]. An assessment of the use of varying social media platforms by different types of users did not result in clear patterns; however, several studies in our review describe engagement of traditionally underserved populations, such as low-income individuals, MSM, and PLWHA on a variety of different social media platforms. Given that these populations are often underaccessed with traditional HIV interventions [75-77], these studies show that social media may be a useful engagement strategy [76]. Our findings also suggest that social media can increase access to both social support and information on HIV prevention and treatment. This increase in access is especially significant for PLWHA who may face barriers to accessing care or prevention resources in-person and for other marginalized groups such as rural populations or young MSM [78,79]. As in other studies [80,81], our review finds that most studies reporting age of platform users engage individuals aged 18 to 40 years, suggesting that health promotion messages using social media may have significant reach within this age group. Given the increasing number of adolescents living with and at risk for HIV [82,83], and the increasing numbers of individuals aged 50 years and older affected by HIV [84], there is a need to explore the feasibility of using social media to communicate about HIV prevention and treatment within these age groups. Additionally, future research could investigate other technologies that may be more acceptable or effective at reaching these populations

Social media platforms have varying designs and features, such as options for anonymity, which can be tailored to meet the needs of target populations and increase use and acceptability of the platform [85,86]. Anonymity allows users to control the information they disclose about themselves, which may allow marginalized populations to feel more comfortable communicating about HIV on social media platforms [87,88]. Most studies in this review do not indicate whether or not individuals communicated using their real names. Interestingly, anonymity is cited as a key benefit and lack of privacy is cited as a key barrier to using social media to communicate about HIV. This dichotomy suggests that social media platforms used to communicate about HIV should allow participants to choose if they would like to remain anonymous in order to facilitate engagement. HIV is a highly stigmatized disease [9,89] in which PLWHA make decisions about disclosure of their HIV status [89,90]. These decisions affect subsequent health behaviors, access to social support, and interactions with sexual partners and social networks [91-93]. Social media platforms may provide PLWHA with an opportunity to anonymously rehearse HIV status disclosure, which may facilitate disclosure in real-world settings [94]. An important next step to using social media to communicate about HIV is to identify which designs best create and facilitate a sense of privacy, confidentiality, and safety.

This review has several limitations. The outcomes of social media communication about HIV prevention and treatment vary greatly. Given the range of outcomes reported, it is difficult to draw any conclusions on how these outcomes relate to the characteristics of users and platforms across studies. This variation represents a significant challenge in surveying the landscape of social media use to communicate about HIV prevention and treatment. Further, the range of literature available on the effectiveness of social media in communication about HIV prevention and treatment is limited. However, this review is a first step to categorizing gaps and trends in the literature in order to identify areas for future research. Despite concerns in the literature about the accuracy of information shared on social media [95-97], this barrier was not reported in our selected studies, and our review was not designed to assess the quality of the information being shared on social media. In addition, the majority of studies selected for this review were at either high or unclear risk of bias for at least one of the bias items. This is a limitation because it is an indicator of the quality of evidence available in the literature and highlights the need for stronger evidence. Lastly, social media use is likely utilized by grassroots and community organizations in HIV communication outside of research contexts; this review did not capture these efforts.

Despite these limitations, our review shows that social media is a promising approach to engage individuals in a dynamic discourse about HIV prevention and treatment, and may allow diverse groups to collaborate on strategies to address the epidemic. Findings from our review are important to design new or leverage pre-existing social media platforms for communication about HIV prevention and treatment, and to illuminate the opportunities for further examination of social media platforms and specific HIV prevention and treatment outcomes.

## Appendix

Multimedia Appendix 1 Studies excluded during full text review.

Multimedia Appendix 2 Summary of selected studies (n=35).

Multimedia Appendix 3 Quality assessment of selected studies.

## Abbreviations

AIDS: acquired immunodeficiency syndrome
HIV: human immunodeficiency virus
MSM: men who have sex with men
N/A: not applicable
PLWHA: people living with HIV/AIDS
PRISMA: Preferred Reporting Items for Systematic Reviews and Meta-Analyses
SES: socioeconomic status
SMS: short message service
UNAIDS: Joint United Nations Programme on HIV/AIDS
