# Supplementary material for: Social Media and HIV: A Systematic Review of Uses of Social Media in HIV Communication
Source: J Med Internet Res. 2015 Nov 2;17(11):e248. doi: 10.2196/jmir.4387 (PMC4642795; doi:10.2196/jmir.4387)
Supplement: Multimedia Appendix 1 [file jmir_v17i11e248_app1.pdf]

Multimedia Appendix 1: Studies Excluded During Full Text Review

EXCLUDED ARTICLES (n=108)

Summary of reasons:

Inclusion Criteria Not Met:

(1) Article does not have a primary focus on communication/interaction about HIV/AIDS (n=42)
(2) Article does not discuss the use of a social media platform to facilitate communication (n=11)
(3) Communication is not between individuals or a group of individuals (n=44)

Exclusion Criteria Met:

(4) Focus on communication/interaction about HIV/AIDS is limited to study implications (n=1)
(5) Article is in a language other than English (n=1)
(6) Article is a commentary (n=4)
(7) Article is a protocol (n=3)
(8) Article is a feature article (i.e. narrative-style, journalistic piece) (n=0)
(9) Article is a letter to the editor (n=0)
(10) There is a primary marketing or advertising focus to the article (n=1)
(11) The social media platform is used for recruitment only (n=0)
(12) The social media platform is used for data collection purposes only (n=1)

LIST OF EXCLUDED ARTICLES

Article does not have a primary focus on communication/interaction about HIV/AIDS (n=42)

1. Kahn JG, Yang JS, Kahn JS. 'Mobile' health needs and opportunities in developing countries. *Health Aff (Millwood)* 2010;29(2):252-258. PMID: 20348069
2. Hooper S, Rosser BR, Horvath KJ, Oakes JM, Danilenko G, Men's INTernet Sex II (MINTS-II) Team. An online needs assessment of a virtual community: what men who use the internet to seek sex with men want in Internet-based HIV prevention. *AIDS Behav* 2008;12(6):867-875. PMID: 18401701
3. Cantudo Cuenca MR, Cantudo Cuenca MD, Morillo Verdugo R. Availability and medical professional involvement in mobile healthcare applications related to pathophysiology and pharmacotherapy of HIV/AIDS. *Eur J Hosp Pharm* 2013;20:356-361. doi:10.1136/ejhpharm-2013-000340
4. Ross MW, Rosser BR, Stanton J. Beliefs about cybersex and Internet-mediated sex of Latino men who have Internet sex with men: relationships with sexual practices in cybersex and in real life. *AIDS Care* 2004;16(8):1002-1011. PMID:15511732
5. Sullivan PS, Khosropour CM, Luisi N, Amsden M, Coggia T, Wingood GM, DiClemente RJ. Bias in online recruitment and retention of racial and ethnic minority men who have sex with men. *J Med Internet Res* 2011;13(2):e38. PMID: 21571632

6. Miller-Nesbitt A. CATIE: Canada's source for HIV and Hepatitis C information. *J Consum Health Internet.* 2013;17(1):95-102. doi:10.1080/15398285.2012.723990
7. Hallett J, Brown G, Maycock B, Langdon P. Changing communities, changing spaces: the challenges of health promotion outreach in cyberspace. *Promot Educ* 2007;14(3):150-154. PMID:18154224
8. Branch K. Computerized sources of AIDS information. *Med Ref Serv Q* 1988;7(4):1-18. PMID:10304127
9. Michael, BD, Geleta D. Development of ClickClinica: a novel smartphone application to generate real-time global disease surveillance and clinical practice data. *BMC Med Infom Decis Mak* 2013:13:70. PMID: 23816161
10. Enah C, Moneyham L, Vance DE, Childs G. Digital gaming for HIV prevention with young adolescents. *J Assoc Nurses AIDS Care* 2013;24(1):71-80. PMID: 22871481
11. Noar SM, Willoughby JF. eHealth interventions for HIV prevention. *AIDS Care* 2012;24(8):945-952. PMID:22519523
12. Burdette SD. Electronic tools for infectious diseases and microbiology. *Can J Infect Dis Med Microbiol* 2007;18(6):347-352. PMID:18978984
13. Khosropour CM, Johnson BA, Ricca AV, Sullivan PS. Enhancing retention of an Internet-based cohort study of men who have sex with men (MSM) via text messaging: randomized controlled trial. *J Med Internet Res* 2013;15(8):e194. PMID: 23981905
14. Young SD, Jaganath D. Feasibility of using social networking technologies for health research among men who have sex with men: a mixed methods study. *Am J Mens Health* 2014;8(1):6-14. PMID: 23407600
15. Levine D, Madsen A, Wright E, Barar RE, Santelli J, Bull S. Formative research on MySpace: online methods to engage hard-to-reach populations. *J Health Commun* 2011;16(4):448-454. PMID: 21391040
16. Loudon M. ICTs as an opportunity structure in Southern social movements: A case study of the Treatment Action Campaign in South Africa. *Information Communication and Society*. 2010;13(8):1069-1098. doi: 10.1080/13691180903468947
17. Vergel N. Impact of body changes on the quality of life of HIV-positive treatment-experienced patients - an online community-based survey. *Antivir Ther* 2008;13(8):A85.
18. Klausner JD, Levine DK, Kent CK. Internet-based site-specific interventions for syphilis prevention among gay and bisexual men. *AIDS Care* 2004;16(8):964-970. PMID: 15511728
19. Mbuagbaw L, Ongolo-Zogo P, Thabane L. Investigating community ownership of a text message programme to improve adherence to antiretroviral therapy and provider-client communication: a mixed methods research protocol. *BMJ Open* 2013;3(6). PMID: 23801710

20. Tikkanen R, Ross MW. Looking for sexual compatibility: Experiences among Swedish men in visiting Internet gay chat rooms. *Cyberpsychol Behav* 2000;3(4):605-616. doi:10.1089/109493100420205.
21. Young SD, Rivers C, Lewis B. Methods of using real-time social media technologies for detection and remote monitoring of HIV outcomes. *Prev Med*. 2014;63:112-115. PMID: 24513169
22. Downing MJ. *Perceptions of risk, sexual behaviors, and HIV prevention in commercial and public sex venues: A study of MSM venue attendees,* ProQuest, UMI Dissertations Publishing; 2010. ISBN 9781124063034
23. Lemay NV, Sullivan T, Jumbe B, Perry CP. Reaching remote health workers in Malawi: baseline assessment of a pilot mHealth intervention. *J Health Commun* 2012;17 Suppl 1:105-117. PMID: 22548604
24. Stokes CE. Representin' in cyberspace: sexual scripts, self-definition, and hip hop culture in Black American adolescent girls' home pages. *Cult Health Sex* 2007;9(2):169-184. PMID: 17364724
25. Sarris R, Sweeton JC. Signing on for health: initiating a computerized health information bulletin board. *J Am Coll Health* 1991;39(4):195-197. PMID: 1995659
26. Reback CJ, Grant DL, Fletcher JB, Branson CM, Shoptaw S, Bowers JR, Charania M, Mansergh G. Text messaging reduces HIV risk behaviors among methamphetamine-using men who have sex with men. *AIDS Behav* 2012;16(7):1993-2002. PMID: 22610370
27. George S, Phillips R, McDavitt B, Adams W, Mutchler MG. The cellular generation and a new risk environment: implications for texting-based sexual health promotion interventions among minority young men who have sex with men. *AMIA Annu Symp Proc* 2012;2012:247-256. PMID: 23304294
28. Allison S, Bauermeister JA, Bull S, Lightfoot M, Mustanski B, Shegog R, Levine D. The intersection of youth, technology, and new media with sexual health: moving the research agenda forward. *J Adolesc Health* 2012;51(3):207-212. PMID: 22921129
29. Schnall R, Okoniewski A, Tiase V, Low A, Rodriguez M, Kaplan S. Using text messaging to assess adolescents' health information needs: an ecological momentary assessment. *J Med Internet Res* 2013;15(3):e54. PMID: 23467200
30. Maher JE, Pranian K, Drach L, Rumptz M, Casciato C, Guernsey J. Using text messaging to contact difficulty-to-reach study participants. *Am J Public Health* 2010;100(6):969-970. PMID: 20395564
31. Wright KB, Rains SA. Weak-Tie Support Network Preference, Health-Related Stigma, and Health Outcomes in Computer-Mediated Support Groups. *J Appl Commun Res* 2013;41(3):309-324. doi: 10.1080/00909882.2013.792435
32. Lunin LF. When you-or your patients-need information on AIDS, databases to search, bulletin boards to access, and hotlines to call. ***J Med Pract Manage*** 1991;7(2):119-124.
33. Mackenzie SL, Kurth AE, Spielberg F, Severynen A, Malotte CK, St Lawrence J, Fortenberry JD. Patient and staff perspectives on the use of a computer counseling

tool for HIV and sexually transmitted infection risk reduction. *J Adolesc Health* 2007;40(6):572.e9-572.e16. PMID: 17531766

34. Zolfo M, Iglesias D, Kiyan C, Echevarria J, Fucay L, Llacsahuanga E, de Waard I, Suàrez V, Llaque WC, Lynen L. Mobile learning for HIV/AIDS healthcare worker training in resource-limited settings. *AIDS Res Ther* 2010;7:35. PMID: 20825677
35. Mo PK, Coulson NS. Online support group use and psychological health for individuals living with HIV/AIDS. *Patient Educ Couns* 2013;93(3):426-432. PMID: 23669152
36. Veinot TC, Campbell TR, Kruger D, Grodzinski A, Franzen S. Drama and danger: the opportunities and challenges of promoting youth sexual health through online social networks. *AMIA Annu Symp Proc* 2011;2011:1436-1445. PMID: 22195207
37. Ralph LJ, Berglas NF, Schwartz SL, Brindis CD. Finding Teens in TheirSpace: Using Social Networking Sites to Connect Youth to Sexual Health Services. *Sex Res Soc Policy* 2011;8:38-49. doi: 10.1007/s13178-011-0043-4
38. Rains SA. The implications of stigma and anonymity for self-disclosure in health blogs. *Health Commun* 2014;29(1):23-31. PMID: 23356432
39. Frost JH, Massagli MP, Wicks P, Heywood J. How the Social Web supports patient experimentation with a new therapy: the demand for patient-controlled and patient-centered informatics. *AMIA Annu Symp Proc* 2008:217-221. PMID: 18999176
40. Brown CH, Mohr DC, Gallo CG, Mader C, Palinkas L, Wingood G, Prado G, Kellam SG, Pantin H, Poduska J, Gibbons R, McManus J, Ogihara M, Valente T, Wulczyn F, Czaja S, Sutcliffe G, Villamar J, Jacobs C. A computational future for preventing HIV in minority communities: how advanced technology can improve implementation of effective programs. *J Acquir Immune Defic Syndr* 2013;63 Suppl 1:S72-84. PMID: 23673892
41. Moody VA. *Descriptive study of internet-based partner services programs for control and prevention of sexually transmitted diseases and the human immunodeficiency virus*, ProQuest, UMI Dissertations Publishing; 2012. ISBN 1267822759
42. Kasatpibal N, Viseskul N, Srikantha W, Fongkaew W, Surapagdee N, Grimes RM. Developing a web site for human immunodeficiency virus prevention in a middle income country: a pilot study from Thailand. *Cyberpsychol Behav Soc Netw* 2012:15(10):560-3. PMID: 23002987

Article does not discuss the use of a social media platform to facilitate communication (n=11)

1. Veinot TC, Meadowbrooke CC, Loveluck J, Hickok A, Bauermeister JA. How "community" matters for how people interact with information: mixed methods study of young men who have sex with other men. *J Med Internet Res* 2013;15(2):e33. PMID: 23428825
2. Hong Y, Li X, Fang X, Lin X, Zhang C. Internet use among female sex workers in China: implications for HIV/STI prevention. *AIDS Behav* 2011;15(2):273-282. PMID: 21082341
3. Pachankis JE, Lelutiu-Weinberger C, Golub SA, Parsons JT. Developing an online health intervention for young gay and bisexual men. *AIDS Behav* 2013;17(9):2986-2998. PMID: 23673791

4. Muessig KE, Pike EC, Fowler B, LeGrand S, Parsons JT, Bull SS, Wilson PA, Wohl DA, Hightow-Weidman LB. Putting Prevention in their pockets: developing mobile phone-based HIV interventions for black men who have sex with men. *AIDS Patient Care STDS* 2013;27(4):211-222. PMID: 23565925
5. Labacher L, Mitchell C. Talk or text to tell? How young adults in Canada and South Africa prefer to receive STI results, counseling, and treatment updates in a wireless world *J Health Commun* 2013;18(12):1465-1476. PMID: 24015829
6. Horvath KJ, Danilenko GP, Williams ML, Simoni J, Amico KR, Oakes JM, Simon Rosser BR. Technology use and reasons to participate in social networking health websites among people living with HIV in the US. *AIDS Behav* 2012;16(4):900-910. PMID: 22350832
7. Wright E, Fortune T, Juzang I, Bull S. Text messaging for HIV prevention with young Black men: formative research and campaign development. *AIDS Care* 2011;23(5):534-541. PMID: 21287416
8. Jaganath D, Gill HK, Cohen AC, Young SD. Harnessing Online Peer Education (HOPE): integrating C-POL and social media to train peer leaders in HIV prevention. *AIDS Care* 2012;24(5):593-600. PMID: 22149081
9. Marhefka SL, Fuhrmann HJ, Gilliam P, Lopez B, Baldwin J. Interest in, concerns about, and preferences for potential video-group delivery of an effective behavioral intervention among women living with HIV. *AIDS Behav* 2012;16(7):1961-1969. PMID: 21947780
10. Shoveller J, Knight R, Davis W, Gilbert M, Ogilvie G. Online sexual health services: examining youth's perspectives. *Can J Public Health* 2012;103(1):14-18. PMID: 22338322
11. Caceres C, Gomez EJ, Garcia F, Chausa P, Guzman J, Del Pozo F, Gatell JM. A home integral telecare system for HIV/AIDS patients. *Stud Health Technol Inform* 2005;114:23-29. PMID: 15923757

Communication is not between individuals or a group of individuals (n=44)

1. Miller CW, Himelhoch S. Acceptability of mobile phone technology for medication adherence interventions among HIV-positive patients at an urban clinic. *AIDS Res Treat* 2013;2013:670525. PMID: 23997948
2. Cornelius JB, St Lawrence JS, Howard JC, Shah D, Poka A, McDonald D, White AC. Adolescents' perceptions of a mobile cell phone text messaging-enhanced intervention and development of a mobile cell phone-based HIV prevention intervention. *J Spec Pediatr Nurs* 2012;17(1):61-69. PMID: 22188273
3. Haberer JE, Kiwanuka J, Nansera D, Wilson IB, Bangsberg DR. Challenges in using mobile phones for collection of antiretroviral therapy adherence data in a resource-limited setting. *AIDS Behav* 2010;14(6):1294-1301. PMID: 20532605
4. Worley R. *Citizen Journalism and Digital Voices: Instituting a Collaborative Process between Global Youth, Technology and Media for Positive Social Change,* ProQuest, UMI Dissertations Publishing; 2011. ISBN 9781124554525
5. Ingersoll K, Dillingham R, Reynolds G, Hettema J, Freeman J, Hosseinbor S, Winstead-Derlega C. Development of a personalized bidirectional text messaging tool for HIV adherence assessment and intervention among substance abusers. *J Subst Abuse Treat* 2014;46(1):66-73. PMID: 24029625

6. Skinner D, Rivette U, Bloomberg C. Evaluation of use of cellphones to aid compliance with drug therapy for HIV patients. *AIDS Care* 2007;19(5):605-607. PMID: 17505920
7. Jennings L, Ong'ech J, Simiyu R, Sirengo M, Kassaye S. Exploring the use of mobile phone technology for the enhancement of the prevention of mother-to-child transmission of HIV program in Nyanza, Kenya: a qualitative study. *BMC Public Health* 2013;13:1131. PMID: 24308409
8. Dowshen N, Kuhns LM, Gray C, Lee S, Garofalo R. Feasibility of interactive text message response (ITR) as a novel, real-time measure of adherence to antiretroviral therapy for HIV+ youth. *AIDS Behav* 2013;17(6):2237-2243. PMID: 23546844
9. Vijaykumar S. *Global health 2.0: Youth, Internet, and the fight against HIV/AIDS,* ProQuest, UMI Dissertations Publishing; 2010. ISBN 1124198504
10. Gordon R, Bjorklund NK, Smith RJ, Blyden ER. Halting HIV/AIDS with avatars and havatars: a virtual world approach to modelling epidemics. *BMC Public Health* 2009;9 Suppl 1:S13. PMID: 19922683
11. Wagenaar BH, Sullivan PS, Stephenson R. HIV knowledge and associated factors among internet-using men who have sex with men (MSM) in South Africa and the United States. *PLoS One* 2012;7(3):e32915. PMID: 22427908
12. Chiasson MA, Hirshfield S, Rietmeijer C. HIV prevention and care in the digital age. *J Acquir Immune Defic Syndr* 2010;55 Suppl 2:S94-S97. PMID: 21406996
13. Holt M, Rawstorne P, Wilkinson J, Worth H, Bittman M, Kippax S. HIV testing, gay community involvement and internet use: social and behavioural correlates of HIV testing among Australian men who have sex with men. *AIDS Behav* 2012;16(1):13-22. PMID: 21213035
14. Flicker S, Goldberg E, Read S, Veinot T, McClelland A, Saulnier P, Skinner H. HIV-positive youth's perspectives on the Internet and e-health. *J Med Internet Res* 2004;6(3):e32. PMID: 15471758
15. Chang LW, Kagaayi J, Arem H, Nakigozi G, Ssempijja V, Serwadda D, Quinn TC, Gray RH, Bollinger RC, Reynolds SJ. Impact of a mHealth intervention for peer health workers on AIDS care in rural Uganda: a mixed methods evaluation of a cluster-randomized trial. *AIDS Behav* 2011;15(8):1776-1784. PMID: 21739286
16. Harris JL, Furberg R, Martin N, Kuhns L, Lewis MA, Coomes C, Williams P, Uhrig JD. Implementing an SMS-based intervention for persons living with human immunodeficiency virus. *J Public Health Manag Pract* 2013;19(2):E9-16. PMID: 23358304
17. Dowshen N, Kuhns LM, Johnson A, Holoyda BJ, Garofalo R. Improving adherence to antiretroviral therapy for youth living with HIV/AIDS: a pilot study using personalized, interactive, daily text message reminders. *J Med Internet Res* 2012;14(2):e51. PMID: 22481246
18. Kurth A, Kuo I, Peterson J, Azikiwe N, Bazerman L, Cates A, Beckwith CG. Information and communication technology to link criminal justice reentrants to HIV care in the community. *AIDS Res Treat* 2013;2013:547381. PMID: 23984054
19. Gagliardo C, Murray M, Saiman L, Neu N. Initiation of antiretroviral therapy in youth with HIV: a U.S.-based provider survey. *AIDS Patient Care STDS* 2013;27(9):498-502. PMID: 23937549

20. Krakower DS, Mimiaga MJ, Rosenberger JG, Novak DS, Mitty JA, White JM, Mayer KH. Limited awareness and low immediate uptake of pre-exposure Prophylaxis among Men Who Have Sex with Men Using an Internet Social networking site. *PLoS One* 2012;7(3):e33119. PMID: 22470438
21. O'Grady L. Meeting health information needs of people with HIV/AIDS: sources and means of collaboration. *Health Info Libr J* 2008;25(4):261-269. PMID: 19076672
22. Muessig KE, Pike EC, Legrand S, Hightow-Weidman LB. Mobile phone applications for the care and prevention of HIV and other sexually transmitted diseases: a review. *J Med Internet Res* 2013;15(1):e1. PMID: 23291245
23. Chib AI. *Network influences in health initiatives: Multimedia games for youth in Peru,* ProQuest, UMI Dissertations Publishing; 2007. ISBN 0549236805
24. Mbuagbaw L, Thabane L, Ongolo-Zogo P. Opening communication channels with people living with HIV using mobile phone text messaging: insights from the CAMPS trial. *BMC Res Notes* 2013;6:131. PMID: 23557081
25. Mitchell JW, Petroll AE. Patterns of HIV and sexually transmitted infection testing among men who have sex with men couples in the United States. *Sex Transm Dis* 2012;39(11):871-876. PMID: 23060078
26. Kinyua F, Kiptoo M, Kikuvi G, Mutai J, Meyers AF, Muiruri P, Songok E. Perceptions of HIV infected patients on the use of cell phone as a tool to support their antiretroviral adherence; a cross-sectional study in a large referral hospital in Kenya. *BMC Public Health* 2013;13:987. PMID: 24143931
27. Khosropour CM, Sullivan PS. Predictors of retention in an online follow-up study of men who have sex with men. *J Med Internet Res* 2011;13(3):e47. PMID: 21745792
28. Moore DJ, Montoya JL, Blackstone K, Rooney A, Gouaux B, Georges S, Depp CA, Atkinson JH, Tmarc Group T. Preliminary evidence for feasibility, use, and acceptability of individualized texting for adherence building for antiretroviral adherence and substance use assessment among HIV-infected methamphetamine users. *AIDS Res Treat* 2013;2013:585143. PMID: 24078868
29. O'Grady LA. *See one, do one, teach one: HIV/AIDS learners participate in communities of practice,* ProQuest, UMI Dissertations Publishing; 2006. ISBN 0494157763
30. Phillips KA, Epstein DH, Mezghanni M, Vahabzadeh M, Reamer D, Agage D, Preston KL. Smartphone delivery of mobile HIV risk reduction education. *AIDS Res Treat* 2013;2013:231956. PMID: 24159383
31. Young SD. Social media technologies for HIV prevention study retention among minority men who have sex with men (MSM). *AIDS Behav* 2014;18(9):1625-1629. PMID: 24062015
32. Lewis MA, Uhrig JD, Bann CM, Harris JL, Furberg RD, Coomes C, Kuhns LM. Tailored text messaging intervention for HIV adherence: a proof-of-concept study. *Health Psychol* 2013;32(3):248-253. PMID: 22545972
33. Furberg RD, Uhrig JD, Bann CM, Lewis MA, Harris JL, Williams P, Coomes C, Martin N, Kuhns L. Technical implementation of a multi-component, text message-based intervention for persons living with HIV. *JMIR Res Protoc* 2012;1(2):e17. PMID: 23612237

34. Harris LT, Lehavot K, Huh D, Yard S, Andrasik MP, Dunbar PJ, Simoni JM. Two-way text messaging for health behavior change among human immunodeficiency virus-positive individuals. *Telemed J E Health* 2010;16(10):1024-1029. PMID: 21087122
35. Theriault N, Bi P, Hiller JE, Nor M. Use of web 2.0 to recruit Australian gay men to an online HIV/AIDS survey. *J Med Internet Res* 2012;14(6):e149. PMID: 23128646
36. Minniear TD, McIntosh EB, Alexander N, Weidle PJ, Fulton J. Using electronic surveys to gather information on physician practices during a response to a local epidemic--Rhode Island, 2011. *Ann Epidemiol* 2013;23(8):521-523. PMID: 23830934
37. Chib A, Wilkin H, Hoefman B. Vulnerabilities in mHealth implementation: a Ugandan HIV/AIDS SMS campaign. *Glob Health Promot* 2013;20(1 Suppl):26-32. PMID: 23549699
38. Waddell RD, Kulig RP. Webcasting: An innovative approach to HIV/AIDS professional training in a rural setting. ***J HIV AIDS Soc Serv*** 2005;4:45-55. doi: 10.1300/J187v04n02_05
39. Chib A, Wilkin H, Ling LX, Hoefman B, Van Biejma H. You have an important message! Evaluating the effectiveness of a text message HIV/AIDS campaign in Northwest Uganda. *J Health Commun* 2012;17 Suppl 1:146-157. PMID: 22548607
40. Lee DM, Fairley CK, Sze JK, Kuo T, Cummings R, Bilardi J, Chen MY. Access to sexual health advice using an automated, internet-based risk assessment service. *Sex Health* 2009;6(1):63-66. PMID: 19254494
41. Marsch LA, Grabinski MJ, Bickel WK, Desrosiers A, Guarino H, Muehlbach B, Solhkhah R, Taufique S, Acosta M. Computer-assisted HIV prevention for youth with substance use disorders. *Subst Use Misuse* 2011;46(1):46-56. PMID: 21190405
42. Hoefman B, Apunyo B. Using SMS for HIV/AIDS education and to expand the use of HIV testing and counseling services at the AIDS information Centre, Uganda. *Mobile Communication Technology for Development (M4D), Kampala, Uganda.* 2010.
43. Curran K, Mugo NR, Kurth A, Ngure K, Heffron R, Donnell D, Celum C, Baeten JM. Daily short message service surveys to measure sexual behavior and pre-exposure prophylaxis use among Kenyan men and women. *AIDS Behav*. 2013;17(9):2977-2985. PMID: 23695519
44. Chang LW, Njie-Carr V, Kalenge S, Kelly JF, Bollinger RC, Alamo-Talisuna S. Perceptions and acceptability of mHealth interventions for improving patient care at a community-based HIV/AIDS clinic in Uganda: a mixed methods study *AIDS Care* 2013;25(7):874-880. PMID: 23452084

Focus on communication/interaction about HIV/AIDS is limited to study implications (n=1)

1. Suwamaru JK. An SMS-based HIV/AIDS education and awareness model for rural areas in Papua New Guinea. *Stud Health Technol Inform* 2012;182:161-169. PMID: 23138091

Article is in a language other than English (n=1)

1. Beier KM, Kuhle LF. Internet and the new media: Perspectives for sexual medicine. *Sexuologie*. 2010;17:139-139-146.

Article is a commentary (n=4)

1. Kuriansky J, Corsini-Munt S. Engaging multiple stakeholders for healthy teen sexuality: Model partnerships for education and HIV/AIDS prevention. In Schroeder E, Kuriansky J, eds. *Sexuality education: Past, present, and future, vol 3: Principles and practices.* Westport: Praeger Publishers/Greenwood Publishing Group; 2009: 311-334. ISBN 978-0-275-99800-4
2. Thirumurthy H, Lester RT. M-health for health behaviour change in resource-limited settings: applications to HIV care and beyond. *Bull World Health Organ* 2012;90(5):390-392. PMID: 22589574
3. Young SD. Recommended guidelines on using social networking technologies for HIV prevention research. *AIDS Behav* 2012;16(7):1743-1745. PMID: 22821067
4. Rossotti R, Gabrielli E, Gervasoni C, Rosso R, Sabbatini F, Uglietti A, Rosso V, Orani A. HIV education and counselling using Facebook: a possible new approach. *J Telemed Telecare* 2012;18(4):239-240. PMID: 22604274

Article is a protocol (n=3)

1. van der Kop ML, Ojakaa DI, Patel A, Thabane L, Kinagwi K, Ekstrom AM, Smillie K, Karanja S, Awiti P, Mills E, Marra C, Kyomuhangi LB, Lester RT. The effect of weekly short message service communication on patient retention in care in the first year after HIV diagnosis: study protocol for a randomised controlled trial (WelTel Retain). *BMJ Open* 2013;3(6). PMID: 23794578
2. Lester RT, Mills EJ, Kariri A, Ritvo P, Chung M, Jack W, Habyarimana J, Karanja S, Barasa S, Nguti R, Estambale B, Ngugi E, Ball TB, Thabane L, Kimani J, Gelmon L, Ackers M, Plummer FA. The HAART cell phone adherence trial (WelTel Kenya1): a randomized controlled trial protocol. *Trials* 2009;10:87. PMID: 19772596
3. Miranda J, Cote J, Godin G, Blais M, Otis J, Gueheneuc YG, Fadel G, Fowler S. An Internet-based intervention (Condom-Him) to increase condom use among HIV-positive men who have sex with men: protocol for a randomized controlled trial. *JMIR Res Protoc* 2013;2(2):e39. PMID: 24132072

Article is a feature article (i.e. narrative-style, journalistic piece) (n=0)

Article is a letter to the editor (n=0)

There is a primary marketing or advertising focus to the article (ie. selling something) (n=1)

1. Bauer R. AIDS information on the Internet (Part 2): Critical Path Internet services. *Crit Path AIDS Proj* 1996;(No 31):27-30. PMID: 11363830

The social media platform is used for recruitment only (n=0)

The social media platform is used for data collection purposes only (n=1)

1. Hirshfield S, Chiasson MA, Joseph H, Scheinmann R, Johnson WD, Remien RH, Shaw FS, Emmons R, Yu G, Margolis AD. An online randomized controlled trial evaluating HIV prevention digital media interventions for men who have sex with men. *PLoS One* 2012;7(10):e46252. PMID: 23071551
