# Supplementary material for: Social Media and HIV: A Systematic Review of Uses of Social Media in HIV Communication
Source: J Med Internet Res. 2015 Nov 2;17(11):e248. doi: 10.2196/jmir.4387 (PMC4642795; doi:10.2196/jmir.4387)
Supplement: Multimedia Appendix 2 [file jmir_v17i11e248_app2.pdf]

Multimedia Appendix 2: Summary of Selected Studies (n=35)

Author, year	Study Method	Type of Social Media Platform	Participants/Sample	Topics of Discussion
Adam et al 2011 [42]	Quantitative	blog	20844 visitors to the website hivstigma.com, 8 bloggers who were gay men	ethical/moral issues, stigma, disclosure, sense of community
Baelden et al 2012 [43]	Qualitative, Quantitative	discussion forum/board	Up to approx. 15000 students at University of the Western Cape	not reported
Brennan et al 1991 [44]	Qualitative	discussion forum/board	19 adults living with HIV/AIDS	sense of community, tangible support/resources, living with HIV, news and events
Broaddus & Dickson-Gomez 2013 [45]	Qualitative	SMS/messaging (individual)	20 African American individuals; 10 men and 10 women	sex, risk/risk reduction, HIV/STI testing
Bull et al 2012 [46]	Quantitative	social media	942 individuals	disclosure, risk/risk reduction, HIV/STI testing, relationships
Coursaris & Liu 2009 [14]	Qualitative	discussion forum/board	approximately 3700 registered members, and anonymous visitors	sense of community, tangible support/resources, HIV/STI facts, HIV/STI testing, living with HIV, news and events
Dean et al 2012 [47]	Qualitative, Quantitative	SMS/messaging (group)	7 pregnant women newly diagnosed with HIV	risk/risk reduction, HIV/STI testing, relationships, living with HIV, reproductive health
Desouza & Jyoti Dutta 2008 [13]	Qualitative	video; discussion forum/board	Members of Saathii e-forum online group	sense of community, tangible support/resources, living with HIV, news and events, advocacy, professional/clinical
Divecha et al 2012 [48]	Quantitative	SMS/messaging (individual); social media	94 urban adolescent and young adult parents	sex, HIV/STI testing
Dunbar et al 2003 [49]	Quantitative	SMS/messaging (individual)	25 individuals living with HIV	risk/risk reduction, living with HIV, news and events, technological issues
Eastham 2011 [50]	Qualitative	blog	14 HIV+ blog authors. Unreported number of blog readers	stigma, disclosure, sense of community, HIV/STI testing, relationships, living with HIV, advocacy
Feldacker et al 2011 [51]	Quantitative	social media to private correspondence	Not specified	not reported

Hightow-Weidman et al 2014 [52]	Quantitative	SMS/messaging (individual), social media to private correspondence	230 Intimate Partner Notification contacts notified; 29 Intimate Partner Notification contacts notified via text messaging	online exposure notification/results
Hildebrand et al 2013 [53]	Qualitative	social media	3,497 young people age 15-29	sense of community, tangible support/resources, living with HIV, professional/clinical
Horvath et al 2013 [15]	Quantitative	SMS/messaging (individual and group)	66 HIV-positive MSMs	sense of community, tangible support/resources, living with HIV, professional/clinical
Ko et al 2013 [12]	Quantitative	social media	369 Internet Popular Opinion Leaders and 499 MSMs from intervention website	risk/risk reduction, HIV/STI testing
Kvasny & Igwe 2008 [54]	Qualitative	blog	Bloggers part of a community of Black bloggers	risk/risk reduction, advocacy
Leon et al 2011 [18]	Qualitative, Quantitative	video, chat (individual and group)	83 HIV+ patients (76 who completed the study)	living with HIV, news and events, professional/clinical
Lester et al 2010 [55]	Qualitative, Quantitative	SMS/messaging (individual)	273 individuals initiating ART for the first time	living with HIV
Lou et al 2006 [56]	Quantitative	discussion forum/board	624 high school and college students in Shanghai	sense of community, sex, risk/risk reduction, relationships, reproductive health
Mo & Coulson 2008 [16]	Qualitative	discussion forum/board	171 visitors to the bulletin board	sense of community, tangible support/resources, HIV/STI facts, risk/risk reduction, living with HIV, advocacy
Moskowitz et al 2009 [57]	Qualitative, Quantitative	chat (individual chat feature of chatroom)	Up to 200 users of Kansas City chatroom on Gay.com	tangible support/resources, HIV/STI facts, sex, risk/risk reduction, HIV/STI testing
Pavlescak 2007 [58]	Qualitative, Quantitative	chat (individual chat feature of chatroom)	304 male clients of chat site for MSM	risk/risk reduction
Pedrana et al 2013 [59]	Qualitative, Quantitative	social media	Members of the public who view the intervention Facebook and YouTube pages	disclosure, sense of community, tangible support/resources, sex, risk/risk reduction, HIV/STI testing
Reid et al 2012 [60]	Qualitative, Quantitative	conferencing	697 health professionals	risk/risk reduction, living with HIV, professional/clinical
Rhodes 2004 [61]	Qualitative	chat (individual chat feature of chatroom)	619 chatters in a chat room for MSM	sense of community, tangible support/resources, sex, risk/risk reduction, HIV/STI testing
Rhodes et al 2010 [62]	Qualitative, Quantitative	chat (individual chat feature of chatroom)	1,851 chatters in a MSM-oriented internet chat room	tangible support/resources, HIV/STI facts, sex , risk/risk reduction, HIV/STI testing

Rice et al 2012 [63]	Quantitative	social media	7 peer leaders, 53 face to face homeless youth, 103 additional youth as friends of the online program	risk/risk reduction
Rothpletz-Puglia et al 2013 [64]	Qualitative and quantitative	discussion forum/board	19 women (who completed)	sense of community, tangible support/resources, risk/risk reduction, living with HIV, advocacy, empowerment
Skrajner et al 2009 [65]	Qualitative, Quantitative	video	23 people living with HIV	sense of community, living with HIV, technological issues
Strand 2011 [17]	Qualitative, Quantitative	blog	6 bloggers living with HIV	living with HIV, news and events, advocacy
Wicks et al 2010 [66]	Quantitative	SMS/messaging (individual and group)	1323 members of PatientsLikeMe communities (including 177 members of HIV community)	living with HIV
Yamauchi 2010 [67]	Qualitative, Quantitative	discussion forum/board, SMS/messaging (individual and group)	45 young people	sense of community, tangible support/resources, HIV/STI facts, sex, risk/risk reduction, relationships, entertainment messages, advocacy, reproductive health
Young & Jaganath 2013 [68]	Qualitative, Quantitative	social media, social media to private correspondence	8 peer leaders, 57 participants. All men who have sex with men.	stigma, sense of community, HIV/STI facts, sex, risk/risk reduction, HIV/STI testing, living with HIV, news and events, advocacy
Zhuang & Bresnahan 2012 [69]	Qualitative, Quantitative	discussion forum/board	275 threads from Users of Chinese online communities	living with HIV, news and events, advocacy, reproductive health
