# Supplementary material for: Social Media and HIV: A Systematic Review of Uses of Social Media in HIV Communication
Source: J Med Internet Res. 2015 Nov 2;17(11):e248. doi: 10.2196/jmir.4387 (PMC4642795; doi:10.2196/jmir.4387)
Supplement: Multimedia Appendix 3 [file jmir_v17i11e248_app3.pdf]

Multimedia Appendix 3: Quality Assessment of Selected Studies

Author, year	(1) methods for selecting study participants	(2) methods for measuring exposure and outcome variables	(3) design-specific source of bias	(4) method of control confounding	(5) statistical methods	(6) other biases (i.e. conflict of interest and disclosure of funding sources)
Adam et al 2011 [42]	-	?	-	+	+	+
Baelden et al 2012 [43]	-	N/A	?	N/A	N/A	-
Brennan et al 1991 [44]	-	-	-	-	+	-
Broaddus & Dickson-Gomez 2013 [45]	+	N/A	+	N/A	N/A	+
Bull et al 2012 [46]	+	+	+	+	+	+
Coursaris & Liu 2009 [14]	+	N/A	+	N/A	N/A	-
Dean et al 2012 [47]	?	+	?	N/A	+	+
Desouza & Jyoti Dutta 2008 [13]	?	N/A	?	N/A	N/A	-
Divecha et al 2012 [48]	+	+	+	-	+	-
Dunbar et al 2003 [49]	?	-	-	-	+	+
Eastham 2011 [50]	+	N/A	+	N/A	N/A	+
Feldacker et al 2011 [51]	N/A	N/A	N/A	N/A	N/A	N/A
Hightow-Weidman et al 2014 [52]	+	+	+	?	+	+
Hildebrand et al 2013 [53]	+	N/A	+	N/A	N/A	+
Horvath et al 2013 [15]	+	+	+	+	+	+
Ko et al 2013 [12]	+	+	?	?	+	+
Kvasny & Igwe 2008 [54]	-	N/A	?	N/A	N/A	-
Leon et al 2011 [18]	+	+	+	+	+	+
Lester et al 2010 [55]	+	+	+	+	+	+
Lou et al 2006 [56]	+	?	+	?	+	+
Mo & Coulson 2008 [16]	?	N/A	+	N/A	N/A	-
Moskowitz et al 2009 [57]	+	?	+	?	?	+
Pavlescak 2007 [58]	+	+	?	?	?	?
Pedrana et al 2013 [59]	+	+	?	?	+	+
Reid et al 2012 [60]	+	?	?	N/A	+	+
Rhodes 2004 [61]	+	N/A	+	N/A	N/A	+
Rhodes et al 2010 [62]	?	+	+	?	+	+

Rice et al 2012 [63]	?	?	?	?	+	+
Rothpletz-Puglia et al 2013 [64]	-	+	-	-	+	+
Skrajner et al 2009 [65]	+	?	?	N/A	+	+
Strand 2011 [17]	+	+	?	N/A	+	-
Wicks et al 2010 [66]	+	?	?	+	+	+
Yamauchi 2010 [67]	?	+	?	+	+	?
Young & Jaganath 2013 [68]	+	+	+	+	+	+
Zhuang & Bresnahan 2012 [69]	+	+	+	?	+	-

Key: Low risk of bias: +; High risk of bias: -; Unclear: ?; Not applicable: N/A
